# THE VERACITY FUNCTION: INTEGRITY AND COMPREHENSIVENESS OF EVIDENCE

**DOI:** 10.2340/jrm.v56.40350

**Published:** 2024-09-30

**Authors:** Antti MALMIVAARA

**Affiliations:** 1 Finnish Institute for Health and Welfare, Mannerheimintie 166, FIN-00270 Helsinki; 2 University of Helsinki, Helsinki; 3 Orthon Orthopedic Hospital, Helsinki, Finland

The mega-trends in health and welfare throughout the world are the ageing of populations and an increasing burden of non-communicable chronic conditions (1). These challenges must be met with scaling up and strengthening of rehabilitation everywhere. Indeed, rehabilitation has been considered the key health strategy of the 21^st^ century (2). And the recent WHO Resolution to strengthen rehabilitation in global health systems is a major step in responding to these worldwide challenges (3).

Cochrane Rehabilitation is a most important effort for gathering and disseminating evidence on the effectiveness of various rehabilitative interventions to clinicians and other decision-makers (4). Currently, one in 11 Cochrane Reviews are on rehabilitation interventions according to the pragmatic inclusion criteria developed by Cochrane Rehabilitation (5).

The most important resource for optimizing patient health benefits is healthcare personnel, as the value is created in the interaction between professionals and patients (6). Beneficial interaction is grounded on competence of healthcare personnel, and on a healthcare system that provides appropriate working conditions and environment for the professionals.

The aim of healthcare is humanitarian: to provide accessible and timely, high-quality (including patient-centredness), effective, and safe services to the population in need in an equitable way and with reasonable costs (6). Due to limited resources, the most cost-effective interventions should usually be prioritized. For advancing the humanitarian role of healthcare, 2 major categories of action must be considered: strengthening integrity in decision-making and improving comprehensiveness of evidence appraisal.

## INTEGRITY

According to the Oxford Dictionary for English language, integrity is the quality of being honest and having strong moral principles. Within medicine integrity concerns the degree of faithfulness to the humanitarian aims of healthcare (7).

The first challenge in decision-making is based on the very nature of human cognition. According to the studies by Tversky and Kahneman we human beings are prone to use intuitive reasoning instead of that based on comprehensive assessment of the evidence (8). This intuitive reasoning, however, may lead to coherent stories rather than rigorous assessments (9).

Other difficulties in maintaining high integrity in decision-making are due to non-cognitive factors, such as personal or group-related interests or incentives, degree of personal independence in the decision-making, preferences based on ideology or even identity as a professional, or on prejudices against equity of population groups (7). A recent literature review provides a comprehensive view of cognitive and other human risks of biases (10).

The humanitarian role of healthcare means that curative, palliative, and rehabilitative interventions should be given their equal share in studies on effectiveness of interventions. However, around two-thirds of randomized controlled trials published in the leading medical journals deal with pharmaceutical interventions, less than one-third relate to other conservative treatments, one- tenth surgical interventions, and only 4% rehabilitation interventions (11). Furthermore, a recent review reveals that the majority of systematic reviews published in the leading medical journals seem to focus on pharmacological interventions, one-third on other conservative treatments, one-tenth on surgical interventions, and none on rehabilitation interventions (12).

## COMPREHENSIVENESS OF EVIDENCE

During the Second World War, Abraham Wald, a mathematician, was given the task to calculate how to safeguard fighting planes from German ground fire (13). The empirical evidence consisted solely of observed hits on the fighting planes that had returned from their operations. He realized that those hits which had led to the downfall of the planes during their missions were absent. This kind of selection bias and incomplete documentation of the evidence occurs regularly in clinical research and practice.

The CONSORT statement for reporting of randomized controlled trials and the PRISMA statement for reporting of systematic reviews and meta-analyses emphasize the importance of comprehensive reporting of patient populations, interventions, and outcomes (14, 15). Despite these recommendations, there are currently major deficiencies in documentation of the characteristics of randomized controlled trials in the original studies, but particularly in systematic reviews and meta-analyses. Due to insufficient documentation of patient selection and characteristics, adherence to interventions, and outcomes related to functioning, the generalizability and applicability of evidence remains limited.

In a recent review of randomized controlled trials published in the 4 leading medical journals, the percentages of adequate documentation of patients’ path before randomization varied from 3% to 33%; characteristics of the healthcare settings from 0% to 75%; comorbid conditions from 25% to 50%; functioning from 42% to 54%, behavioural factors from 25% to 58%, environmental factors from 3% to 25%, and inequity-related factors from 28% to 68%; co-interventions from 6% to 25%; and reasons for dropping out of follow-up from 39% to 100% (11).

The reporting and consequently the applicability of evidence from systematic reviews and meta-analyses in the 4 leading medical journals is much poorer than that of RCTs. In a recent study, only 4 out of 115 systematic reviews reported on the included RCTs patients’ eligibility criteria, contents of the interventions, and the primary outcomes. None of the systematic reviews assessed patient selection, one-third reported disorder-specific clinical features, while comorbid conditions and patients’ behavioural factors were reported even less. Functioning was recorded in only 3%, inequity-related factors in 9%, and environmental factors in none of the reviews. Adherence to interventions were reported in 7%, co-interventions in 2%, and crossovers to other interventions in none of the reviews. Share of patients in the follow-up was reported in only 8%, and adequacy of statistical analyses in only 3% of the reviews. Despite the latter, a meta-analysis was undertaken in 90% of the reviews. This means that the outcome values were entered from the original studies to the meta-analyses without checking whether they were based on a sound statistical analysis (12). However, there is evidence that significant deficiencies in statistical analyses of RCTs occur frequently (16).

The cornerstone of evidence-based medicine is validity of the original studies. Therefore, a comprehensive assessment of all relevant validity criteria should be undertaken. Unfortunately, the Cochrane Collaboration Handbook core validity criteria are limited to 7 items (17). Moreover, these items focus on double-blinded study design and lack major validity criteria that are relevant in particular for open study designs, and especially for trials on rehabilitation. Instead of using the core validity criteria, a comprehensive set of 13 Cochrane validity items is needed (18).

The poor reporting in systematic reviews must change. The clinically essential features of patient selection and characteristics, interventions, and outcomes must be reported comprehensively in line with the PRISMA recommendations. Reporting of eligibility criteria, contents of the interventions, and distinction of primary and secondary outcomes is not enough. Also, patient selection, characteristics of patients, adherence to the inventions, shares of crossovers, co-interventions, and all outcomes must be reported. The lacking items must be pointed out because *it is as important to acknowledge the gaps in information as to use the data that are available in order to make sound inferences regarding the applicability of the evidence*. For a comprehensive assessment of evidence, a method has been developed (19). This method can be used both for randomized controlled trials (RCTs) and observational effectiveness studies, benchmarking controlled trials (BCTs), and also systematic reviews for comprehensive data extraction from the original studies.

## THE VERACITY FUNCTION

Reducing bias in medical decision-making from interests other than those related to the humanitarian aims of healthcare involves 2 major categories of action: increasing integrity and comprehensiveness of appraisal of evidence.

The quest for high integrity and comprehensiveness of assessment of evidence are interrelated. High integrity asks about objective assessment of the whole evidence, and comprehensive assessment shows deficiencies that challenge prejudices. High integrity within the realm of healthcare means prioritization based on the benefit that will be created for patients and the overall population.

Comprehensiveness of assessment means a thorough description of the characteristics related to the selection and characteristics of the population, interventions, and outcomes documented in the original studies and a list of those characteristics that remained outside documentation. This also concerns efforts to contain costs by avoiding treatments that are considered of no or of only small value. The uncertainty of evidence and inability to predict the overall cost-effectiveness consequences may decrease the real-world gains. Thus, sometimes better cost-effectiveness is gained by doing less and sometimes by doing more, while often we are not able to predict what the consequences will be (9).

**Fig. 1:**
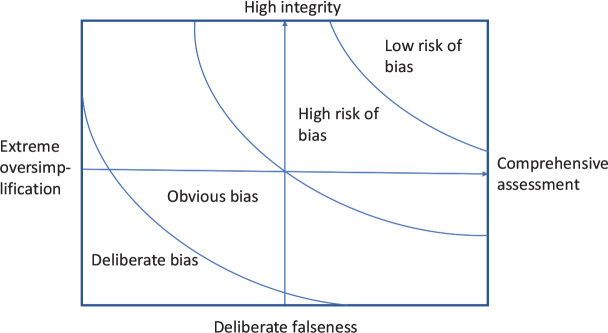
The veracity function: integrity and comprehensiveness of assessment of evidence.

Fig. 1 illustrates the potential for avoiding decision-making bias, the veracity function between integrity and comprehensiveness of assessment. On the y-axis the scale covers the whole spectrum from very high integrity to deliberate falseness. On the x-axis the scale extends from the most comprehensive assessment to the most extreme oversimplification. High-quality scientific research can be found in the area of low risk of bias. Lower quality research is accompanied by high risk of bias. Within the area of obvious bias, experts and methodologists in the field can usually agree that it is not possible to make interpretations based on the available information. The perilous area of extreme oversimplification and deliberate falseness is counterproductive to any constructive pursuit in any society.

The mission in advancing the humanitarian aim of healthcare, both in research and in clinical practice, is always towards the right upper corner of Fig. 1. The veracity function guides the appraisal of current evidence towards low risk of bias.

In conclusion, to optimally promote health and well-being of populations, the humanitarian role of all actions at system and clinical level should prioritized. This calls for avoidance of human risk of bias, i.e., high integrity, and comprehensive assessment of the evidence by all decision-makers.

The practical consequences of the veracity function are twofold: (*i*) avoidance of human risk of bias (increasing integrity) in clinical research and practice and in health policy, and (*ii*) pursuing comprehensive documentation of all pertinent factors when documenting research findings and when making clinical and health policy decisions. Integrity in decisions leads to more equal prioritization for curative, palliative, and rehabilitative interventions. Comprehensive documentation of selection and characteristics of patients, interventions, and outcomes both in the study protocol and in the actual study, as well as appraisal of all the validity items, is a necessity for optimal decision-making.

The veracity function is suggested as a conceptual framework for promoting the humanitarian goals of healthcare.
